# Effects of ambient air pollutants on ARDS incidence and outcome: a narrative review

**DOI:** 10.1186/s13613-023-01182-1

**Published:** 2023-09-13

**Authors:** Laëtitia Gutman, Vanessa Pauly, Laurent Papazian, Antoine Roch

**Affiliations:** 1 Assistance Publique - Hôpitaux de Marseille, Hôpital Nord, Médecine Intensive Réanimation, Chemin Des Bourrely, 13015 Marseille, France; 2 Faculté de Médecine, Centre d’Etudes et de Recherches Sur Les Services de Santé et qualité de vie EA 3279, Aix-Marseille Université, 13005 Marseille, France; 3 Unité d’Analyse Des Données de Santé, Assistance Publique, Hôpitaux de Marseille, 13005 Marseille, France; 4 Médecine Intensive Réanimation, Centre Hospitalier de Bastia, 20600 Bastia, Corsica France

**Keywords:** Acute respiratory distress syndrome, Environmental risk factor, Particulate matter, Nitrogen dioxide, Ozone, Air pollutant

## Abstract

**Background:**

Exposure to air pollutants promotes inflammation, cancer, and mortality in chronic diseases. Acute respiratory distress syndrome (ARDS) is a common condition among intensive care unit patients and is associated with a high mortality rate. ARDS is characterized by significant lung inflammation, which can be replicated in animal models by acute exposure to high doses of various air pollutants. Recently, several clinical studies have been conducted in different countries to investigate the role of chronic or acute air pollutant exposure in enhancing both ARDS incidence and severity.

**Results:**

Chronic exposure studies have mainly been conducted in the US and France. The results of these studies suggest that some air pollutants, notably ozone, nitrogen dioxide, and particulate matter, increase susceptibility to ARDS and associated mortality. Furthermore, their impact may differ according to the cause of ARDS. A cohort study conducted in an urbanized zone in China showed that exposure to very high levels of air pollutants in the few days preceding intensive care unit admission was associated with an increased incidence of ARDS. The effects of acute exposure are more debatable regarding ARDS incidence and severity.

**Conclusion:**

There is a likely relationship between air pollutant exposure and ARDS incidence and severity. However, further studies are required to determine which pollutants are the most involved and which patients are the most affected. Due to the prevalence of ARDS, air pollutant exposure may have a significant impact and could be a key public health issue.

## Introduction

During the twentieth century, industrialization (including road traffic) significantly increased air pollutant concentration and exposure [1]. The World Health Organization (WHO) regularly publishes guidelines on air quality and the fight against air pollutants [2]. In 2021, the WHO estimated that over 10% of the world's population lived in areas where air quality guidelines were not being followed. Exposure to air pollutants is known to increase cancer incidence by promoting chronic inflammation [3–5] and affects life expectancy and mortality related to diabetes, cardiovascular, and chronic respiratory diseases [6–8].

Numerous studies suggest a link between air pollutant exposure and the onset of chronic respiratory diseases [9–11]. The onset of asthma in children has been repeatedly associated with exposure to both indoor and outdoor pollutants during pre- and post-natal childhood and with cigarette smoke exposure [9–12]. The severity and frequency of acute asthma crises were also correlated with outdoor air pollutant exposure [10, 11]. Additionally, several studies have found an association between chronic obstructive pulmonary disease (COPD) exacerbation and acute exposure to outdoor air pollutants [13, 14]. Chronic exposure to outdoor air pollutants during childhood (including antenatal exposure) may also be a risk factor for developing COPD in adulthood [15]. Lastly, a prospective study revealed that chronic exposure to O_3_, NO_2_, and PM_2.5_ was associated with increased emphysema lesions [16]. However, although pollution has been suggested to increase incidence and severity of community-acquired pneumonia [17, 18], less is known about the ability of pollution to promote acute severe diseases.

Acute respiratory distress syndrome (ARDS) is a clinical syndrome characterized by severe hypoxemia and respiratory distress related to non-cardiogenic pulmonary edema, necessitating intensive care unit (ICU) admission and mechanical ventilation in the most severe forms [19]. While ARDS can result from various pathological conditions such as sepsis, pneumonia, or severe trauma, the common pathway among all ARDS causes is significant lung inflammation.

Air pollutants have been shown to induce lung inflammation and are, therefore, used in animal models to mimic ARDS [20]. There is also some evidence suggesting that chronic or acute exposure to outdoor air pollutants may increase the susceptibility of patients at risk of developing ARDS and may affect their outcome. To our knowledge, no study has specifically addressed the link between indoor air pollutant exposure and ARDS incidence. Therefore, we will focus our review solely on outdoor ambient populational air pollutants, hereafter designated as air pollutants. This review will discuss animal and clinical studies related to the role of outdoor air pollutants in promoting ARDS onset and severity. We will also address potential areas for future research on this specific topic.

### Air pollution and ARDS: two major problems potentially linked?

The air we breathe is primarily composed of 78% nitrogen (N) and approximately 21% oxygen (O_2_) [30]. The remaining 1% consists of air pollutants, which can be classified as gases, such as nitrogen dioxide (NO_2_), ozone (O_3_), sulfur dioxide (SO_2_), carbon monoxide (CO), and carbon dioxide (CO_2_), or liquid or solid particles suspended in the atmosphere. All these air pollutants are emitted by various sources, including industries, transportation, domestic activities, and agriculture.

Air pollutants can be divided into two categories depending on their production sources: primary pollutants are directly produced by nature or human activities, such as emissions from fireplaces or manufacturing processes, while secondary pollutants result from the interaction of pollutants with each other or with weather conditions (for example, sun radiation contributing to the formation of ozone) [31]. NO_2_ is released during combustion, with transportation being the main source of emissions. O_3_ is a secondary pollutant resulting from complex interactions between primary pollutants such as NO_2_ and sunlight. Therefore, O_3_ levels mainly depend on solar activity and are not directly correlated with industrial or transport activity. Particulate matter (PM) is classified based on its aerodynamic diameter and is divided into three categories: less than 0.1 µm (PM_0.1_), less than 2.5 µm (PM_2.5_, which includes PM_0.1_), and less than 10 µm (PM_10_, which includes PM_2.5_) [32]. PM is a heterogeneous category of air pollutants containing chemically different particles such as black carbon, sand, or storm dust. Determining the primary cause of PM production is challenging as it can be generated by fuel combustion, pollens, and photochemical reactions with other pollutants, making them primary or secondary pollutants. Moreover, PM is still inhomogeneous despite the size classification, as PM composition varies temporally and geographically due to diverse production sources and meteorological changes [33, 34]. Nonetheless, the combustion of fossil fuels, represented by industrial and domestic activities like inhabitants' fireplaces, remains a significant source of PM emissions in multiple countries. Additionally, for PM_10_, agriculture is also a notable contributor to emissions. Table 1 compiles the results from a recent French national report on 2019 air pollutant sources for primary pollutants, providing more details on emission sources [31].

**Table 1:** Air pollutant sources of emission, based on the Citepa report, France [13]

Air pollutant	Transport	Industry: manufacture	Industry: Energy	Domestic activity	Agriculture	Nature	Secondary pollutant
PM_2.5_	+ +	+ +	−	+ + + +	+	NA	Yes
PM_10_	+ +	+ + +	−	+ + +	+ + +	NA	Yes
NO_2_	+ + + +	+	+	+	+	−	No
O_3_	NC	NC	NC	NC	NC	NC	Yes
SO_2_	±	+ + + +	+ + +	+	−	−	No
CO	+	+ + +	±	+ + + +	+	−	No
CO_2_	+ + + +	+ +	+	+ +	±	−	No

Average results for French territory in 2019. In percentage of French emission. - : < 1% ; ± : < 5%; + : 5 to 15 %; ++ : 15 to 25 %; +++ :25 to 35 %; ++++ : > 35%

Nature refers to soil erosion, pollens, biomass fires, volcanic eruptions, dust sand etc.; NA: No value available, however, depending on the site and the atmospheric condition, may be a major transitory factor; NC: not concerned

Air pollutants are associated with morbidity and/or mortality in various diseases. In a retrospective observational study conducted in the United States (US), Pope et al. [6] estimated that a decrease of 10 µg/m^3^ in PM_2.5_ could be responsible for a 0.61-year increase in life expectancy. The large European ELAPSE project [7] found that PM_2.5_, NO_2_, and black carbon were associated with increased global, cardiovascular, respiratory, and diabetes-related mortality rates. However, despite such studies [8], they could not identify a threshold below which air pollutant exposure could be considered harmless. Several studies suggest that air pollutant may promote lung infectious diseases. In 2010, Neupane et al*.* [17] conducted a case report study in elderly people, suggesting that chronic exposure to NO_2_ and PM_2.5_ increases hospitalization for community-acquired pneumonia. In the USA, acute exposure to air pollutant has been shown to be a risk factor for community-acquired pneumonia [18]. Several studies also found an association between air pollutant exposure and COVID-19 incidence [35, 36] and severity [36, 37].

Among respiratory illnesses, ARDS poses a particular challenge for care and research. Although ARDS may have different causes, such as sepsis, pneumonia, or severe trauma, the common pathway between all ARDS causes is inflammatory dysregulation. After host aggression, inappropriate inflammatory responses induce lung leukocyte accumulation and coagulation dysregulation, increasing endothelial and epithelial permeability. In 2014, the LUNG SAFE (Large observational study to UNderstand the Global impact of Severe Acute respiratory FailurE) [38] international survey reported that ARDS accounted for 10.4% of all ICU admissions and affected 23.4% of patients who underwent invasive mechanical ventilation. The ICU mortality rate of patients with ARDS ranges from 34.9% to 46.1% [38]. In a recent national French study, a crude incidence of 24.6 per 100,000 person-years and a hospital mortality rate of 51.2% were reported for ARDS [39].

Animal models have demonstrated that exposure to air pollutants can induce alveolar injury and lung vascular inflammation by triggering oxidative stress in alveolar cells, thereby promoting the recruitment of inflammatory cells and the production of cytokines [40, 41]. As a result, acute exposure to high doses of various air pollutants can lead to an acute lung injury resembling ARDS in animal models, exhibiting clinical, radiological, and histopathological features. In a rat model, inhalation of fire-drill waste, known as white smoke, primarily composed of zinc chloride, hexachloroethane, oxides, and carbon dust, induced typical ARDS, with the mortality rate increasing with exposure dose [42]. Moreover, in rats, exposure to 3 ppm of O_3_ also led to alveolar edema, hypoxemia, and decreased lung compliance [43]. The duration of O_3_ exposure in this study was correlated with the severity of histopathological damage, resulting in a complete ARDS pattern. Leiphrakpam et al. conducted studies in which they induced ARDS in rats and pigs by exposing them to wood smoke inhalation [41, 44]. In both studies, the smoke was at ambient temperature, ruling out burning lesions as the cause of ARDS. The animals exhibited radiological, histological, and clinical features characteristic of ARDS. The smoke used in these experiments consisted of high concentrations of PM_2.5_ (i.e., 135 µg/m^3^ in the rat model).

These animal models, however, do have certain limitations. Firstly, smoke inhalation is a specific model of exposure to high concentrations of indoor air pollutants (e.g., from heating and cooking habits) rather than a model of outdoor air pollutant exposure. Secondly, the levels and composition of PM_0.1_ and PM_10_ have been shown to depend on numerous factors, such as the type of stove used, airflow, burning temperature, and the type of wood burned [45]. These factors can limit the generalizability of these indoor pollutant models. Finally, it is essential to interpret animal studies with caution because they do not fully replicate the complex cocktail effect of various air pollutants present in the human environment. Additionally, animals used in these studies are not exposed to other potential susceptibility factors for ARDS that humans may encounter. As a result, the direct translation of findings from animal studies to the human population requires careful consideration and additional research.

In addition to well-known risk factors that directly cause lung injury and contribute to ARDS, various other factors have been suggested to increase susceptibility to developing ARDS in at-risk patients [46]. These factors include chronic alcohol exposure [47], blood group characteristics [21], and genetic factors [48, 49]. Some studies have also examined cigarette smoke exposure as a susceptibility factor for developing ARDS [28, 29], yielding diverse results depending on the cause of ARDS, indicating that smoke exposure might prime lung injury in smokers.

### Effects of exposure to air pollutants on ARDS incidence in humans

There is no consensus on the definition of chronic or acute exposure to air pollutants. In the literature, exposure is considered chronic if it lasts from 6 months [26] to 5 years [22, 23]. On the other hand, acute exposure is generally defined as ranging from 1 day to 6 weeks [23, 25]. It is important to consider that, although splitting acute and chronic exposure makes physiological sense, people acutely exposed to air pollution also often undergo chronic exposure.

### Chronic exposure

Several studies have suggested that chronic exposure to air pollutants increases the incidence of ARDS in ICU-admitted patients (Table 2). Most of these studies have been conducted in the US.

**Table 2:** Concentration of air pollutants in studies on the role of chronic exposure on the incidence of ARDS

	Lag	PM_2.5_	PM_10_	NO_2_		O_3_	SO_2_	CO
	Years	µg/m^3^	µg/m^3^	µg/m^3^	ppb	ppb	ppb	mg/m^3^
WHO STANDARD		5	15	10	5.3	30.6^c^	NA	NA
Ware^b^ 2016 [22] USA	1, 3, 5	13.2		29.0^c^	15.4	51.5	2.7	0.68
Reilly^b^ 2018 [23] USA	1, 2, 3	12.2		34.0^c^	18.1	47.1	3.6	0.28
Rhee^a^ 2019 [26] USA	0.5	10.8				39.1		
Gutman^a^ 2022 [24] France	1, 2, 3	9.1	19.6	13.1	7.0^x^	62.9^c^		
Reilly^b^ 2023 [27] USA	5	10.9	19.2	29.0^c^	15.5	46.7	2.03	0.26

PM2.5: Particulate matter less than 2.5 μm in aerodynamic diameter; PM10: Particulate matter less than 10 μm; NO_2_: Nitrogen dioxide; O_3_: Ozone, SO_2_: suffer dioxide; CO: Carbon monoxide; ppb: part per billion; ppm: part per million

^a^Expressed as mean; ^b^Expressed as median; ^c^Represents a conversion used to compare the units between studies. The formula for air pollutant were used assuming a temperature of 25°C and an atmospheric pressure of 1013 hPa

WHO standard refers to guidelines from the 2021 update on WHO air quality guidelines. Annual values are reported on those guidelines, except O_3_ where peak season is reported. No air quality guideline is available on SO_2_ and CO chronic exposure but only on 24-hour exposure

For instance, Ware et al. [22] examined 1558 patients with risk factors for ARDS admitted to the ICU in multiple states. Daily measurements of O_3_, NO_2_, SO_2_, PM_2.5_, and PM_10_ were obtained from the Environmental Protection Agency's Aerometric Information Retrieval System. Patient addresses were geocoded, and distances to all monitors were calculated. Daily pollutant exposures were estimated by the inverse-distance-squared weighted average of daily levels from monitors within a 50 km radius. Additionally, a three-year long-term exposure was estimated using average pollutant levels for the 3 years before ICU admission. The study found that the incidence of ARDS increased with increasing O_3_ exposure, being 28% in the lowest exposure quartile versus 32%, 40%, and 42% in the second, third, and fourth quartiles, respectively. The correlation between O_3_ exposure and the incidence of ARDS was strongest in trauma patients, who represented 30% of this cohort. The authors also observed that NO_2_ exposure was correlated with ARDS incidence in the mono-pollutant model with NO_2_ but not in the bi-pollutant model, which included O_3_. This discrepancy might be explained chemically, as NO_2_ and O_3_ levels are inversely correlated, with NO_2_ interacting with other air pollutants and promoting the spread of O_3_. Finally, the authors found that O_3_ was a risk factor to develop ARDS only in current cigarette smokers. This finding that cigarette smoking potentiates the effects of air pollution highlights the need to assess smoking exposure in air pollutant studies on ARDS.

Reilly et al. [23] investigated 996 severe trauma patients, of whom 24% developed ARDS and were admitted to an ICU in Pennsylvania between 2005 and 2015. The region of the study is more urbanized than the one explored in the study by Ware et al. [22], which can, at least in part, explain higher NO_2_ and lower O_3_ levels. The results were notably adjusted for various factors, including the month of enrollment, toxic habits (alcohol use and smoking history), type of trauma, total transfusion in the ICU, pulmonary contusion as a cause of ARDS, and social statistics. This study's long-term (3-year) exposures to O_3_, NO_2_, SO_2_, CO, and PM_2.5_ were significantly associated with the occurrence of ARDS. These results remained consistent even after conducting several sensitivity analyses.

Rhee et al. [26] assessed the incidence of ARDS among US Medicare beneficiaries aged ≥ 65 years from 2000 to 2012 in the USA. The study determined the average levels of PM_2.5_ and O_3_ exposure during the warm season of the same year based on patients’ addresses. The authors categorized ARDS into three etiologies: severe trauma, pneumonia, and sepsis. In single-pollutant models adjusted for socioeconomic and demographic factors, as well as an estimator of smoking, increased exposure to O_3_ over the year was found to be strongly correlated with a higher incidence of ARDS in the subgroup of severe trauma patients. However, the association between PM_2.5_ exposure and ARDS in severe trauma patients was not statistically significant. Interestingly, the rise in PM_2.5_ exposure was significantly correlated with an increase in the incidence of ARDS in sepsis patients and to a slightly lesser extent in pneumonia patients but no longer showed a correlation with ARDS in the subgroup of severe trauma patients.

In a recent retrospective cohort study, Gutman et al. [24] investigated the association between chronic exposure to PM_2.5_, PM_10_, NO_2_, O_3_, and ARDS incidence in a French region (Provence-Alpes-Cote-d’Azur, including Marseille and Nice areas), which had a population of 5 million inhabitants in 2020. This European region is characterized by lower PM and NO_2_ levels compared to the US studies [22, 23] due to its less industrialized nature, resulting in higher O_3_ levels. The study found that an increase in PM_2.5_ and PM_10_ of one standard deviation (i.e., 0.7 and 2.9 µg/m^3^) over a year was associated with a rise in ARDS incidence rate of 1.207 (95% CI 1.145; 1.390) and 1.168 (95% CI 1.083; 1.259), respectively, considering all causes of ARDS. The results remained consistent when considering a 3-year and 2-year average exposure. Regarding NO_2_, the results were inconsistent, as only 1-year and 2-year chronic exposures were related to ARDS incidence. Notably, O_3_ chronic exposure was not associated with ARDS incidence in this study. It is worth mentioning that severe trauma accounted for only 3.9% of the ARDS etiologies in this cohort.

Reilly et al. [27] recently published a cohort study conducted in Pennsylvania, including 1858 patients with sepsis admitted to the ICU, of whom 41% developed ARDS within 6 days following sepsis onset. The study revealed that short-term (3-day) and long-term (5-year) exposures to SO_2_, NO_2_, and PM_2.5_ were associated with an increased risk of ARDS. Exposure to PM_2.5_, PM_10_, NO_2_, O_3_, SO_2_, and CO was monitored using daily levels from monitors within 50 km of subjects' residences, with the ZIP code mean exposure serving as an estimation of real air pollutant exposure for each patient. It is important to note that the association between PM_2.5_ and NO_2_ 5-year exposure and ARDS incidence was found to be linear, further strengthening the link between air pollutant exposure and ARDS incidence. However, the study did not provide precise data regarding hobbies, displacement habits, or indoor air pollutant exposure.

Existing research suggests that specific air pollutants can increase the risk of ARDS in patients already at risk, although the effects may differ among pollutants. Additionally, the impact of pollutants on ARDS incidence may vary depending on the cause of the disease.

### Acute exposure

Some cases of ARDS occurring after high exposure to pollutants have been reported [50]. However, in the Penn Trauma Cohort [23], acute exposure to air pollutants during the 3 days preceding severe trauma was not found to be associated with the incidence of ARDS. The results remained consistent for PM_2.5_, NO_2_, O3, or CO. Nevertheless, a non-linear association was observed for SO_2_.

In China, Lin et al. [25] conducted a study on the incidence of ARDS in relation to exposure to air pollutants in Guangzhou, the third-largest city in China, known for very high levels of acute air pollutants. To describe acute exposure, the researchers examined different time lags of air pollutant exposure up to 5 days before admission. They studied both the averaged cumulative exposure to air pollutants before admission from day 0 to day -t and the unique lag "t" day exposure to air pollutants before admission (i.e., the average exposure on the unique "t" day before admission). The study found that the averaged cumulative exposure to PM_1_, PM_2.5_, and PM_10_ during the 0 to 1 and 0 to 3 days before admission were correlated with ARDS incidence, but not the 0- to 5-day averaged cumulative exposure. Additionally, they observed a significant association with the lag 0 and lag 1 exposure but not for the lags 2, 3, 4, or 5 days of exposure. The interpretation of this data type is complex, but the results support an acute and rapid effect of PM_1_, PM_2.5_, and PM_10_ on ARDS incidence. However, it is important to note that the high levels of pollutants in this study exceeded WHO guidelines, limiting the generalizability of the results to other countries with lower pollutant exposure levels.

Reilly et al. [27] recently conducted a prospective cohort study and demonstrated an association between PM_2.5_ and SO_2_ exposure in the 6-week and 3-day periods before sepsis, respectively. Only NO_2_ exposure in the 3-day period was found to be correlated with an increased incidence of ARDS. To our knowledge, this represents the first study in a Western country at this moderate level of air pollutant exposure to establish a relationship between acute air pollutant exposure and ARDS incidence.

Chinese and North American standards are shown in Table 3.

**Table 3:** Concentrations of the air pollutants in the studies related to the effects of acute exposure on the incidence of ARDS

	Lag	PM_2.5_	PM_10_	NO_2_	O_3_	SO_2_	CO
	Days (D), weeks (W)	µg/m^3^	µg/m^3^	µg/m^3^	ppb	ppb	ppm
WHO STANDARD		15	45	25	100	40	3.5^c^
Reilly^b^ 2018 [23] USA	3D, (5W)	12.2		34.0^c^	92.4^c^	9.4^c^	0.28
Lin^a^ 2018 [25] China	0, 3, 5D	Around 50	Around 75				
Reilly^b^ 2023 [27] USA	3D, (6W)	9.4	18.6	12.6	45.2	1.13	0.24

PM2.5: Particulate matter less than 2.5 μm in aerodynamic diameter; PM10: Particulate matter less than 10 μm; NO_2_: Nitrogen dioxide; O_3_: Ozone; SO_2_: suffer dioxide; CO: Carbon monoxide; ppb: part per billion; ppm: part per million

^a^Expressed as mean; ^b^Expressed as median; ^c^Represents a conversion used to compare the units between studies. The formula for air pollutant was used assuming a temperature of 25 °C and an atmospheric pressure of 1013 hPa

WHO standard refers to guidelines from the 2021 update on WHO air quality guidelines. 24-hour 99th percentiles are reported on those guidelines, except for O_3_ presented as average of daily maximum 8-hour mean O_3_ concentration

If 2 lags were available, the one in parenthesis is not reported

### Exposure to air pollutants and ARDS mortality

In 2000, Rush et al. [51] conducted a national retrospective observational study examining O_3_ and PM_2.5_ exposure and hospital mortality in ARDS patients in the US. The study collected all reported cases of ARDS using the National Inpatient Sample and correlated city exposure to O_3_ with mortality. The researchers compared the 15 cities with the highest air pollutant levels to the 15 cities with the lowest levels. Patients treated in highly polluted areas showed higher ARDS-associated mortality rates (odds ratio 1.13 [95% CI 1.09; 1.16]), even after adjusting for age, sex, a comorbidity index, and renal replacement therapy. This national study was the largest conducted in a Western country. However, its main limitation was the assumption that the ambient air pollutant level in the county of the treating hospital was a good surrogate for individual exposure. Since the median surface of a US county is around 1600 km^2^ [52], it varies greatly according to the state, with Western and southern states having larger counties. Rush et al. [51] were unable to differentiate air pollutant exposure for two patients living in the same county. For example, a rural patient who had never left the countryside and an urban patient living and working in a highly polluted city was considered to have the same air pollutant exposure if treated in the same hospital.

In the French study [24], conducted in a large region, increased chronic exposure to PM_2.5_ was found to be associated with higher 90-day mortality in ARDS patients. This study is the first of its kind in Europe, but it has some limitations, including its retrospective design and the absence of certain parameters such as obesity and cigarette smoke exposure. Despite these limitations, this exploratory study offers valuable insights into understanding the relationship between air pollutant exposure and mortality in ARDS.

**Fig. 1:**
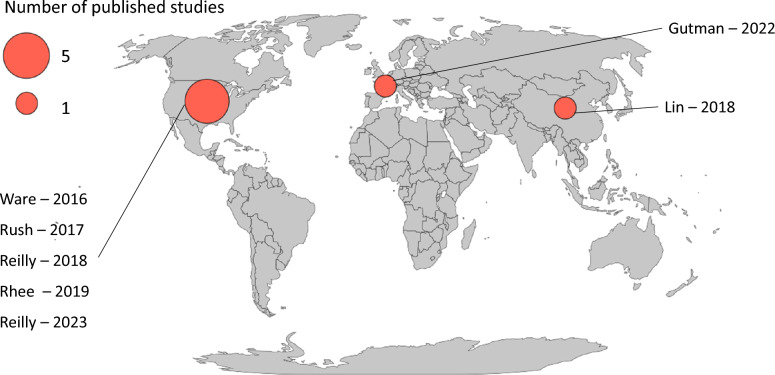
Geographical distribution of the main studies on the impact of air pollutant acute or chronic exposure on ARDS incidence and/or mortality

The geographical distribution of studies on acute and chronic exposure to air pollutants and ARDS is summarized in Fig. 1.

### Future research priorities

The possible relationship between air pollutant exposure and ARDS incidence and severity warrants in-depth investigation. This is because ARDS is a syndrome caused by multiple risk factors, and it is possible that different types of pollutants at varying levels may influence each ARDS sub-population differently, such as severe trauma, sepsis, or pneumonia. While chronic exposure to O_3_ appears to increase ARDS incidence in severe trauma patients, there is a lack of other prospective studies focusing on sepsis and pneumonia patients in relation to air pollutant exposure. Further research is needed to better understand the specific effects of different pollutants on various ARDS subgroups.

Second, most studies have relied on monitoring stations to measure pollutant levels. While some more precise studies have access to the exact location of the patient’s home, to date, no prospective or retrospective study has been able to precisely identify the exposure of each individual patient. Notably, existing research has primarily focused on outdoor air pollutant exposure, overlooking the specific indoor air pollutant exposure that varies with individual habits [45, 53]. The ideal model would involve using personal monitors [54] on patients to measure their individual exposure, but this approach is impractical for large-scale prospective research on ARDS. As an alternative, researchers could consider monitoring the exact exposure of each patient by studying the duration spent in different activities (e.g., work, hobbies, and home) and the average exposure to air pollutants during these activities. Additionally, a more detailed exploration of occupational habits, especially regarding PM and the risk of workplace exposure (e.g., dust, sand, drilling activities) and home exposure related to heating methods, is essential. Regarding acute exposure to air pollutants, factors such as humidity, atmospheric temperature, and pressure [54] can significantly influence the effective level of air pollutant exposure. The lack of evidence linking acute exposure to air pollutants and ARDS may also stem from our current inability to accurately capture the effective dose of pollutants for each patient. Advancements in measuring techniques and data collection methodologies may help improve our understanding of this relationship in the future.

Third, to our knowledge, no validated multi-pollutant indicator specific to respiratory diseases and ARDS has been thoroughly evaluated. In various multi-exposure fields, relying solely on mono-pollutant models may not be sufficient to identify the actual air pollutant risk factors. This crucial aspect must be thoroughly investigated because the absence of a validated multi-pollutant indicator could, in part, explain the challenges in establishing a robust relationship between ARDS and air pollutant exposure.

## Conclusion

The association between chronic exposure to various air pollutants and ARDS incidence remains consistent across clinical studies conducted on different continents. However, further research is needed to validate and corroborate these findings in various countries and under different levels of exposure.

Studies on the effects of acute exposure to air pollutants are currently lacking. The presence of various air pollutants and the absence of a precise definition for what constitutes acute exposure pose significant challenges for future research. The interactions between air pollutants and the lungs are likely complex, contributing to the cocktail effect of different substances and the duration of exposure. To advance our understanding, prospective studies should be conducted with precise definitions of both indoor and outdoor air pollutant exposures. Since acute and chronic air pollutant exposures are often concomitant, future studies should investigate potential “priming” by chronic exposure followed by a “triggering” effect by acute high dose exposure.

Considering the global nature of air pollution, international multicentric studies are essential for future research, as each geographic area has its own unique levels and combinations of exposure to pollutants. Moreover, to comprehend the complex association between air pollutants and ARDS, studies focusing on each specific ARDS etiology should be conducted, allowing for a better understanding and differentiation of the health impacts based on the cause of ARDS and the population affected.

Given the high prevalence and severity of ARDS, air pollutant exposure may have a significant impact and is likely to be a crucial public health issue that demands attention and further investigation.

## Data Availability

Not applicable.

## Abbreviations

ARDS: Acute respiratory distress syndrome
CO: Carbon monoxide
CO_2_: Carbonate dioxide
COPD: Chronic Obstructive Pulmonary Disease
ICU: Intensive care unit
NO_2_: Nitrogen dioxide
O_3_: Ozone
PM: Particulate matter
SO_2_: Suffer dioxide
US: United States
WHO: World Health Organization
